# Spinel-Encapsulated Ni-Rich Cathodes for Enhanced Thermal Safety: Unraveling the Decomposition Kinetics and Interfacial Reconstruction

**DOI:** 10.3390/nano16030183

**Published:** 2026-01-29

**Authors:** Linjie Xie, Huiqi Sun, Jiawei Dou, Juncheng Jiang, Chen Liang

**Affiliations:** 1 School of Safety Science and Engineering, Changzhou University, Changzhou 213164, China; b22200837007@smail.cczu.edu.cn (L.X.);; 2 College of Safety Science and Engineering, Nanjing Tech University, Nanjing 211816, China; 3 State Key Laboratory of Fire Science, University of Science and Technology of China, Hefei 230026, China

**Keywords:** Ni-rich layered cathode, LiNi_0.5_Mn_1.5_O_4_ spinel coating, thermal safety, interface chemistry, spinel-layered heterostructure, decomposition kinetics

## Abstract

High-energy Ni-rich layered cathodes are critical for next-generation lithium-ion batteries yet remain limited by severe interfacial degradation and thermal vulnerability under high-voltage operation. In this work, a robust spinel-layered heterostructure is constructed by encapsulating LiNi_0.8_Co_0.1_Mn_0.1_O_2_ (NCM811) with a LiNi_0.5_Mn_1.5_O_4_ (LNMO) spinel shell via a scalable sol–gel route. Structural characterizations confirm that the coating maintains the secondary-particle architecture, while X-ray photoelectron spectroscopy reveals a chemically reconditioned interface, achieved by the scavenging residual lithium species and suppressing of rock-salt-like surface reconstruction. Consequently, the optimized 4 wt% LNMO@NCM811 electrode demonstrates significantly enhanced high-voltage (2.8–4.4 V) stability, maintaining 41.84% of its initial capacity after 200 cycles compared to only 15.75% for the pristine sample. Crucially, thermogravimetric-differential scanning calorimetry (TG-DSC) uncovers the kinetic origin of this safety improvement: the spinel shell alters the thermal decomposition pathway, delaying the 10% mass loss temperature (T_10%_) from 515.2 °C to 716.6 °C and suppressing the total exothermic heat release from 208.3 J g^−1^ to 81.5 J g^−1^. Collectively, these results demonstrate that the co-free spinel encapsulation is a dual-functional strategy to simultaneously stabilize surficial chemistry and intrinsically enhance the thermal safety of Ni-rich cathodes for carbon-neutral energy storage applications.

## 1. Introduction

Nickel-rich layered oxides, represented by LiNi_0.8_Co_0.1_Mn_0.1_O_2_ (NCM811), have emerged as leading cathode candidates for next-generation lithium-ion batteries owing to their high specific capacity of approximately 200 mAh g^−1^ [1] and reduced reliance on costly cobalt [2]. Recent comprehensive assessments by research groups in Europe and North America have further emphasized the urgency of reducing cobalt content not only for cost reduction but also for ethical and supply chain sustainability [3,4]. However, the commercial deployment of NCM811 is hampered by pronounced surface instabilities and thermal vulnerability, particularly under high cut-off voltages exceeding 4.3 V [5,6,7]. The highly reactive surfaces of Ni-rich particles are susceptible to moisture and CO_2_ [8], spontaneously forming insulating residual lithium species such as LiOH and Li_2_CO_3_ [9]. These alkaline residues not only induce slurry gelation but also trigger severe parasitic reactions with the electrolyte, generating gaseous by-products and resistive rock-salt-like surface layers [10,11]. Recent comprehensive reviews have highlighted that the electrochemical performance of Ni-rich cathodes is intrinsically limited by crystal defects and surface instabilities [12]. To mitigate these issues, transition-metal-oxide coatings have emerged as a pivotal design strategy to stabilize the interface and suppress phase transitions [13], yet the specific kinetic mechanisms governing these heterostructures require further elucidation. Leading studies from international laboratories have highlighted that these interfacial side reactions are a universal challenge, exacerbated significantly at high states of charge [14]. Recent reviews have highlighted that these parasitic side reactions are particularly exacerbated at high cutoff voltages, necessitating robust interfacial protection strategies [15]. As documented by Dahn et al. and other prominent groups, these micro-cracks act as channels for fresh electrolyte infiltration, accelerating the degradation of the particle interior [16]. In addition, the abrupt anisotropic lattice contraction and expansion associated with the high-voltage phase transition from the hexagonal H_2_ phase, a highly delithiated hexagonal phase, to the hexagonal H_3_ phase, a more deeply delithiated hexagonal phase, generate immense internal strain, leading to intergranular cracking [17,18]. The newly formed cracks expose the particle interior to further electrolyte attack, accelerating capacity decay and, in extreme cases, contributing to catastrophic thermal runaway [19,20]. It is worth noting that LiNiCoAlO_2_ (NCA), another prominent high-nickel cathode, typically demonstrates superior capacity retention and structural stability compared to NCM counterparts, attributed to the strong Al–O bonding that mitigates lattice collapse [21]. However, NCM811 remains highly attractive due to the earth-abundance and cost-effectiveness of manganese compared to cobalt-rich formulations. The challenge lies in the fact that Mn^4+^ in NCM provides less robust structural pinning than Al^3+^ in NCA during deep delithiation.

To mitigate these interfacial degradation phenomena, surface engineering has been widely adopted as a standard protocol [22,23]. A variety of coating materials, ranging from inert metal oxides such as Al_2_O_3_ and ZrO_2_ to phosphates and polymeric films, have been employed to construct a physical barrier between the cathode and the electrolyte [24]. While such traditional passivation layers can suppress side reactions to some extent, they often suffer from poor ionic conductivity and limited electronic conductivity [25]. The resulting increase in interfacial impedance compromises lithium-ion transport and undermines the rate capability and power performance of the battery [26,27]. To address this, the construction of functional heterostructures, in which a structurally compatible and ionically conductive shell encapsulates the layered core, has emerged as a more promising strategy to balance interfacial protection with charge-transport requirements [28,29].

Among the various candidates for such functional shells, the spinel LiNi_0.5_Mn_1.5_O_4_ (LNMO) is particularly attractive [30,31]. The LNMO phase possesses a robust three-dimensional framework with interconnected interstitial pathways for fast Li^+^ transport and exhibits a high operating voltage plateau of around 4.7 V, enabling it to function as an electrochemically active “armor” rather than a passive, insulating layer [32,33]. Unlike layered oxides, the spinel structure exhibits superior stability against thermal and electrochemical stress, a property that has been extensively verified in recent structural modification studies of LNMO [34]. Although spinel coatings have been explored to improve electrochemical performance [35], most existing studies remain limited to empirical optimization and qualitative safety assessments. The intrinsic correlation between the heterostructure design and the thermal decomposition kinetics of Ni-rich cathodes remains largely unexplored. Specifically, it is unclear whether the coating fundamentally alters the decomposition reaction pathway or merely imposes a physical delay. However, the fundamental understanding of how such spinel-layered heterostructures influence thermal safety remains relatively superficial [36]. Most reports qualitatively attribute enhanced safety to a generic physical isolation effect, without fully resolving how the surface heterostructure modifies the intrinsic thermal decomposition kinetics of Ni-rich cathodes [37,38]. It remains unclear whether the coating merely slows down existing decomposition reactions through changes in kinetic parameters such as activation energy and pre-exponential factor, or whether it alters the dominant reaction model and decomposition pathway [39,40]. However, most existing studies remain limited to empirical optimization and qualitative safety assessments. Addressing this knowledge gap is crucial for the rational design of Ni-rich cathodes with intrinsically improved thermal safety, rather than relying on purely empirical optimization [41,42,43,44]. To bridge this gap, this work presents a comprehensive study on unraveling the kinetic origin of thermal safety enhancement in LNMO@NCM811. Distinct from traditional inert coatings, we demonstrate that the electrochemically active spinel shell functions through a dual mechanism: (1) chemically scavenging alkaline residues to recondition the interface, and (2) mechanically clamping the bulk lattice to suppress the H2→H3 phase transition. Uniquely, we apply model-free kinetic analysis (TG-DSC) to quantify the change in activation energy and reaction models, providing new mechanistic insights beyond simple physical isolation.

Herein, we report a scalable sol–gel strategy to construct a robust spinel-layered heterostructure by conformally wrapping NCM811 with a functional LNMO shell. This architecture is designed as a dual-action interfacial modifier: chemically, the LNMO shell scavenges detrimental alkaline surface residues and suppresses rock-salt-like surface reconstruction; mechanically, its rigid spinel framework exerts a clamping effect that moderates the high-voltage H_2_→H_3_ phase transition and alleviates micro-strain accumulation during deep delithiation. Benefiting from this integrated interfacial design, the LNMO@NCM811 cathode delivers markedly improved high voltage cyclability and enhanced thermal stability under aggressive operating conditions.

Furthermore, systematic thermogravimetric-differential scanning calorimetry combined with model-free non-isothermal kinetic analysis reveals that the robust spinel shell modifies the thermal decomposition pathway. This work establishes a mechanistic link between interfacial engineering and intrinsic thermal safety, offering a viable route to reconcile the energy–safety trade-off and facilitating the sustainable deployment of high-energy Ni-rich cathodes.

## 2. Materials and Methods

Lithium acetate, nickel acetate and manganese acetate (analytical grade, >99%) and citric acid (≥99.5%) were purchased from Aladdin Reagent Co., Ltd. (Shanghai, China) and used as precursors for the sol–gel synthesis of LiNi_0.5_Mn_1.5_O_4_ (LNMO). Anhydrous ethanol and deionized (DI) water were used as solvents in the sol–gel synthesis and subsequent coating of NCM811. Commercial NCM811 powder was supplied by Rongbai Technology Co., Ltd. (Ningbo, China). According to the supplier’s specifications, the material undergoes rigorous purification with sulfur and sodium impurities strictly controlled below 500 ppm. All chemicals were used as received and stored in a dry, dark environment to minimize moisture uptake and light-induced degradation [45]. LiNi_0.5_Mn_1.5_O_4_ (LNMO) was synthesized by a citrate-assisted sol–gel method. In a typical procedure, 0.273 g of lithium acetate, 0.312 g of nickel acetate and 0.924 g of manganese acetate were dissolved in 300 mL of deionized water under magnetic stirring. Subsequently, 100 mL of an aqueous citric acid solution containing 0.192 g of citric acid was added dropwise. The pH of the solution was adjusted to 6.5 using ammonium hydroxide, and the mixture was stirred at 65 °C and 800 rpm until a homogeneous gel formed. The gel was dried in a vacuum oven at 110 °C for 15 h and then calcined at 500 °C for 6 h in a tube furnace under air (80 mL min^−1^). The obtained powder was pulverized using a mortar and pestle to ensure homogeneity and used as the LNMO precursor for the subsequent surface-coating process.

LNMO-coated NCM811 composites were prepared by a wet-impregnation method followed by high-temperature annealing. The as-synthesized LNMO powder and commercial NCM811 powder were co-dispersed in anhydrous ethanol and stirred at 1200 rpm for 8 h at 25 °C to promote the adsorption and deposition of LNMO particles onto the surface of NCM811 secondary particles. The suspension was then dried in a vacuum oven at 100 °C for 12 h, and the dried mixture was subsequently calcined in a tube furnace at 850 °C for 12 h in an ambient air atmosphere. While Ni-rich cathodes are typically sensitive to air, air calcination was employed to promote the crystallization of the Mn-based spinel phase. The mass ratio of LNMO to NCM811 was adjusted so that the LNMO content was 1, 2 and 4 wt%, affording the samples denoted as 1 wt% LNMO@NCM811, 2 wt% LNMO@NCM811 and 4 wt% LNMO@NCM811, respectively. Pristine NCM811 without LNMO coating was used as a reference sample.

The pristine and LNMO-coated NCM811 samples were systematically characterized to correlate their structural and interfacial features with electrochemical performance. Crystal structures were examined by X-ray diffraction (XRD, Rigaku SmartLab, Tokyo, Japan) using Cu K*α* radiation (λ = 1.5406 Å) over a 2θ range of 10–80° at a scan rate of 4° min^−1^. Rietveld refinement was carried out to evaluate the lattice parameters and phase purity using GSAS-II software (version 5798). The refined structural models were based on the hexagonal *α*-NaFeO_2_ structure (R3m space group) for the NCM811 phase and the cubic spinel structure (Fd3m space group) for the LNMO phase. During the refinement, a Chebyshev polynomial function was used to fit the background, and a pseudo-Voigt function was employed to model the peak profiles. Key parameters, including lattice constants were iteratively refined until convergence. The reliability of the fitting results was assessed based on the weighted profile R-factor (R_wp_) and the goodness-of-fit (GOF), ensuring statistically significant structural solutions. Surface morphologies were observed by scanning electron microscopy (SEM, Zeiss Gemini 500, Oberkochen, German). X-ray photoelectron spectroscopy (XPS, Thermo Scientific K-Alpha, Thermo Fisher Scientific, Waltham, MA, USA) was employed to determine the surface elemental composition and oxidation states of the cathodes after cycling. Al K*α* radiation was used as the excitation source [46]. Survey spectra were collected at a pass energy of 150 eV with a step size of 1.0 eV, and high-resolution spectra at 50 eV with a step size of 0.1 eV. Data analysis and peak deconvolution were performed using Avantage software (Thermo Fisher Scientific, v5.948, Build 06186).

The thermal behavior of the cathodes was analyzed using thermogravimetric-differential scanning calorimetry (TG-DSC, NETZSCH STA, NETZSCH, Selb, German) [47]. After the lithium-ion coin cells had undergone 200 charge–discharge cycles and were finally charged to 4.4 V, the cells were disassembled in a high-purity argon-filled glovebox. The recovered electrodes were repeatedly rinsed with dimethyl carbonate (DMC), centrifuged, and then dried under argon for 12 h to remove residual electrolyte [48]. Approximately 3 ± 0.5 mg of the dried sample was placed in an alumina crucible and heated from room temperature to 800 °C at a rate of 10 °C min^−1^ under flowing argon. Simultaneous TG-DSC measurements recorded the mass loss and heat flow to evaluate the thermal decomposition characteristics and high-temperature stability of the cathode materials [49]. CR2032 coin cells were assembled to evaluate the electrochemical performance of the pristine and LNMO-coated NCM811 samples. Cathode electrodes were prepared by casting a slurry containing the active material, polyvinylidene fluoride (PVDF) binder and acetylene black conductive additive (mass ratio 8:1:1) in N-methyl-2-pyrrolidone (NMP) onto aluminum foil current collectors [50], followed by drying under vacuum at 110 °C for 12 h. The dried electrodes were punched into 14 mm disks, giving an active-material areal loading of 5.2 ± 0.3 mg cm^−2^ for all cathodes. Lithium metal foil was used as both the counter and reference electrode. The electrolyte consisted of 1 M LiPF6 dissolved in a mixture of ethylene carbonate and ethyl methyl carbonate (EC/EMC = 3:7, *v*/*v*), and a Celgard 2500 microporous polypropylene membrane (Celgard, LLC, Charlotte, NC, USA) was used as the separator. Cell assembly was carried out in an argon-filled glovebox, and all cells were rested at 25 °C for 12 h prior to testing. Galvanostatic charge–discharge tests were performed on a NEWARE CT-4008 system (NEWARE, Shenzhen, China) in a voltage range of 2.8–4.4 V (vs. Li^+^/Li) at 25 °C. The cells were first subjected to three formation cycles at 0.1 C, followed by 200 cycles at 0.5 C.

## 3. Results and Discussion

### 3.1. Phase Identification and Interfacial Surface Chemistry

**Figure 1:**
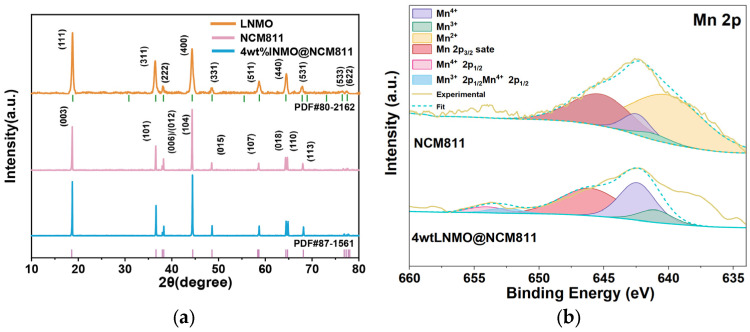
Structural and chemical characterization. (**a**) XRD patterns; (**b**) Mn 2p XPS spectra.

The crystallographic structural integrity of the synthesized materials was first evaluated by XRD. As shown in Figure 1a, the diffraction pattern of the synthesized LNMO matches well with the cubic spinel structure (space group Fd$\overset{\text{-}}{3}$m) and JCPDS No. 80-2162, while the commercial NCM811 exhibits well-defined peaks indexed to the hexagonal α-NaFeO_2_ layered structure (space group R$\overset{\text{-}}{3}$m) and JCPDS No. 87-1561. For the 4 wt% LNMO@NCM811 composite, the pattern is dominated by the characteristic reflections of the layered NCM811 core. The absence of secondary phases indicates that the wet-chemical coating process does not induce bulk structural degradation of the Ni-rich cathode. The absence of distinct spinel diffraction peaks in the composite is attributed to the low mass fraction of the LNMO shell and the overlapping of its main reflections with the intense peaks of the bulk layered oxide.

To probe the surface chemical states and verify the protective role of the coating under electrochemical stress, XPS analyses were conducted on the electrodes recovered after cycling [51]. Figure 1b presents the high-resolution Mn 2p spectra. The spectra were deconvoluted into three components corresponding to Mn^4+^, Mn^3+^, and Mn^2+^ species. While both samples exhibit a dominant Mn^4+^ character typical of delithiated states, a significant difference is observed in the concentration of unstable trivalent manganese. The pristine NCM811 surface contains a notable fraction of Mn^3+^ (7.2%), which is prone to disproportionation reaction (2Mn^3+^ → Mn^4+^ + Mn^2+^) and Jahn–Teller distortion, leading to transition metal dissolution and structural collapse. In stark contrast, the 4 wt% LNMO@NCM811 sample shows a markedly suppressed Mn^3+^ content of only 2.3%. This reduced Mn^3+^ fraction suggests that the robust LNMO spinel shell effectively serves as a physical barrier, mitigating surface reconstruction and inhibiting the formation of degradation-active species during high-voltage cycling.

### 3.2. Evolution of the Layered Structure and Lattice Strain

**Figure 2:**
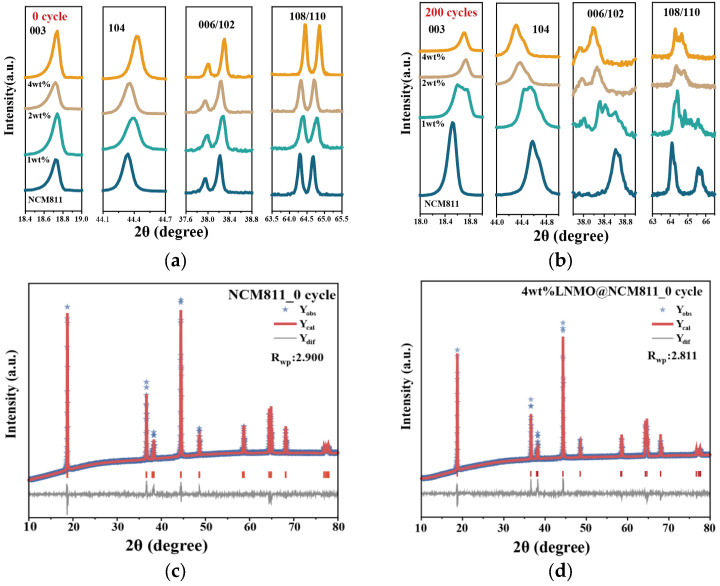

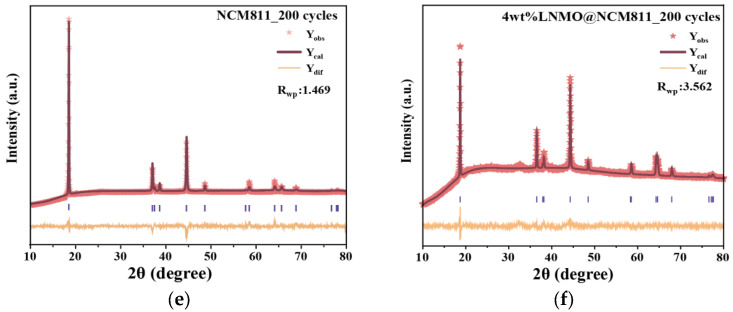
XRD characterization. (**a**) Selected magnified regions of fresh electrodes; (**b**) selected magnified regions after 200 cycles; (**c**) Rietveld plot of fresh NCM811; (**d**) Rietveld plot of fresh 4 wt% sample; (**e**) Rietveld plot of cycled NCM811; (**f**) Rietveld plot of cycled 4 wt% sample.

To elucidate the stabilization mechanism of the LNMO shell, the structural evolution of the cathodes was analyzed by XRD and Rietveld refinement before and after 200 cycles. Figure 2a compares the magnified reflections of the fresh electrodes. All samples show a clear separation of the 003 and 104 reflections and distinct splitting of the 006/102 and 108/110 doublets, confirming a well-ordered *α*-NaFeO_2_ layered structure with the R$\overset{\text{-}}{3}$m space group. The peak positions of the coated electrodes are close to those of pristine NCM811, indicating that the LNMO treatment does not alter the bulk layered framework in the pristine state. Consistently, the Rietveld refinements at 0 cycle in Figure 2c,d reproduce the experimental patterns well, giving agreement factors of R_wp_ = 2.900 for pristine NCM811 and R_wp_ = 2.811 for 4 wt% LNMO@NCM811. Quantitatively, Table 1 shows that 4 wt% LNMO@NCM811 presents a higher I(003)/I(104) ratio of 1.395 compared with 1.274 for pristine NCM811, explicitly indicating suppressed Li/Ni mixing via the spinel coating, together with a slightly lower micro-strain at the beginning of cycling.

After 200 cycles, pronounced structural divergence becomes evident. As shown in Figure 2b, pristine NCM811 exhibits broadened reflections and weakened splitting of the 006/102 and 108/110 doublets, consistent with increased lattice distortion and a progressive loss of layered order. The corresponding refinement in Figure 2e provides a reasonable description of the pattern with R_wp_ = 1.469 and indicates a contraction along the *c* direction and a decrease in the *c*/*a* ratio to 4.935, as summarized in Table 2. In contrast, 4 wt% LNMO@NCM811 retains more obvious peak splitting and comparatively sharper profiles in Figure 2b, and its cycled pattern is also captured by refinement in Figure 2f with R_wp_ = 3.562. The refined lattice parameters show a larger *c*-axis of 14.3475 Å and a higher *c*/*a* ratio of 5.055, indicating suppressed *c*-axis contraction and better preservation of the layered periodicity. Notably, the refined micro-strain of the coated sample after cycling is slightly higher than that of the pristine electrode. This retained strain in the coated sample paradoxically indicates preserved structural integrity, whereas the pristine sample likely underwent stress relaxation via irreversible particle pulverization and micro-cracking. Overall, the peak evolution in Figure 2a,b, together with the Rietveld fits in Figure 2c–f and the refined parameters in Table 1 and Table 2, demonstrates that an adequately formed LNMO coating, especially at 4 wt%, slows the degradation of layered order in NCM811 during long-term cycling, even though it does not completely prevent strain buildup.

### 3.3. Influence of LNMO Coating on NCM811 Particle Morphology and Structural Integrity

**Figure 3:**
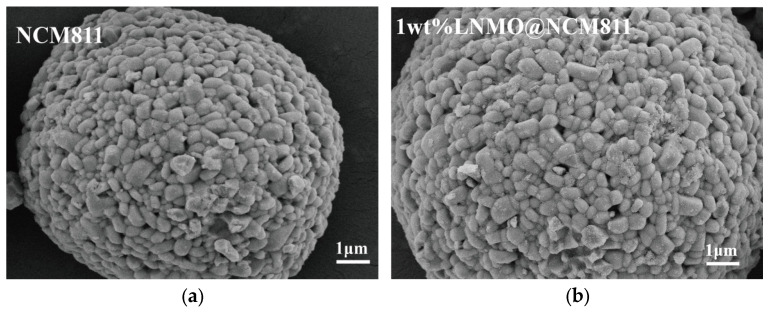

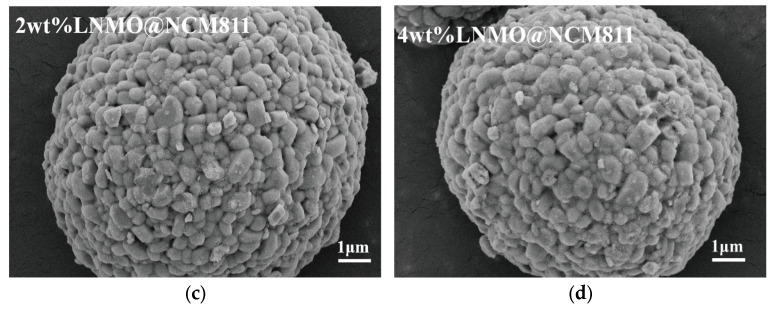
SEM images of LNMO-coated and pristine NCM811. (**a**) Pristine NCM811; (**b**) 1 wt% LNMO@NCM811; (**c**) 2 wt% LNMO@NCM811; and (**d**) 4 wt% LNMO@NCM811.

The influence of the LNMO coating on the particle morphology was examined by SEM, as shown in Figure 3. All samples exhibit the typical pseudo-spherical secondary particles of NCM811, composed of closely packed primary grains with submicrometre sizes. No fractured spheres, severe cracking, or collapsed agglomerates are observed, confirming that the sol–gel coating and subsequent annealing process do not compromise the structural integrity of the NCM811 secondary-particle architecture. Distinct morphological evolution is observed in the surface texture. Since all samples were prepared using the same batch of commercial NCM811 and the coating layer thickness is negligible compared to the secondary particle diameter, the intrinsic particle size distribution remains consistent across the pristine and coated samples. The apparent size variations observed in the SEM images are therefore attributed to the random selection of particles within the limited field of view rather than synthesis-induced discrepancies. The pristine NCM811 shows a relatively smooth surface, where the outlines of individual primary grains and the grain boundaries are clearly distinguishable (Figure 3d). For a more detailed elemental distribution analysis, SEM and EDS mappings of the pristine NCM811 are provided in Supplementary Information, Figure S2. In contrast, the LNMO-coated samples display rougher and more compact surfaces. For the 1 wt% and 2 wt% LNMO@NCM811 electrodes, discrete granular features appear on top of the primary grains (Figure 3a,b), attributed to the deposition of LNMO nanoparticles. When the loading is increased to 4 wt%, the surface becomes continuously covered; the boundaries between primary particles become blurred, and inter-particle voids are partially filled (Figure 3c) see Supplementary Information, Figure S1. SEM and EDS elemental mapping of the 4wt% LNMO@NCM811 are shown in Supplementary Information, Figure S3, which further highlight the elemental distribution on the surface. Crucially, the absence of independent agglomerates suggests a high affinity between the spinel coating and the layered host, implying that the wet-impregnation followed by annealing successfully anchors the shell without phase segregation. The selection of the 4 wt% loading was determined based on an optimization balance between surface coverage and electrochemical kinetics. A loading lower than 4 wt% typically results in a discontinuous coating (island-like), which fails to fully isolate the NCM811 surface from the electrolyte. Conversely, a loading higher than 4 wt% creates an excessively thick shell that, despite the ionic conductivity of LNMO, introduces additional diffusion impedance and reduces the overall specific capacity. Therefore, the 4 wt% dosage was identified as the optimal engineering point. Crucially, these morphological features observed in SEM directly underpin the enhanced electrochemical behavior presented later. First, the uniform and conformal nature of the 4 wt% LNMO shell acts as a rigid “clamping layer”. It mechanically restrains the abrupt anisotropic volume expansion of the NCM811 secondary particles during cycling, thereby suppressing intergranular cracking. This morphological integrity directly correlates with the significantly improved capacity retention observed in the cycling tests. Second, the texture of the coating, characterized by interconnected spinel nano-crystallites, establishes a robust 3D conduction network. By hermetically sealing the reactive Ni-rich surface, the coating prevents the continuous growth of a resistive CEI layer. Consequently, this morphological design facilitates rapid interfacial charge transfer, serving as the structural origin for the superior rate capability and reduced polarization.

### 3.4. Surface Chemical Reconstruction and Electronic State Analysis

**Figure 4:**
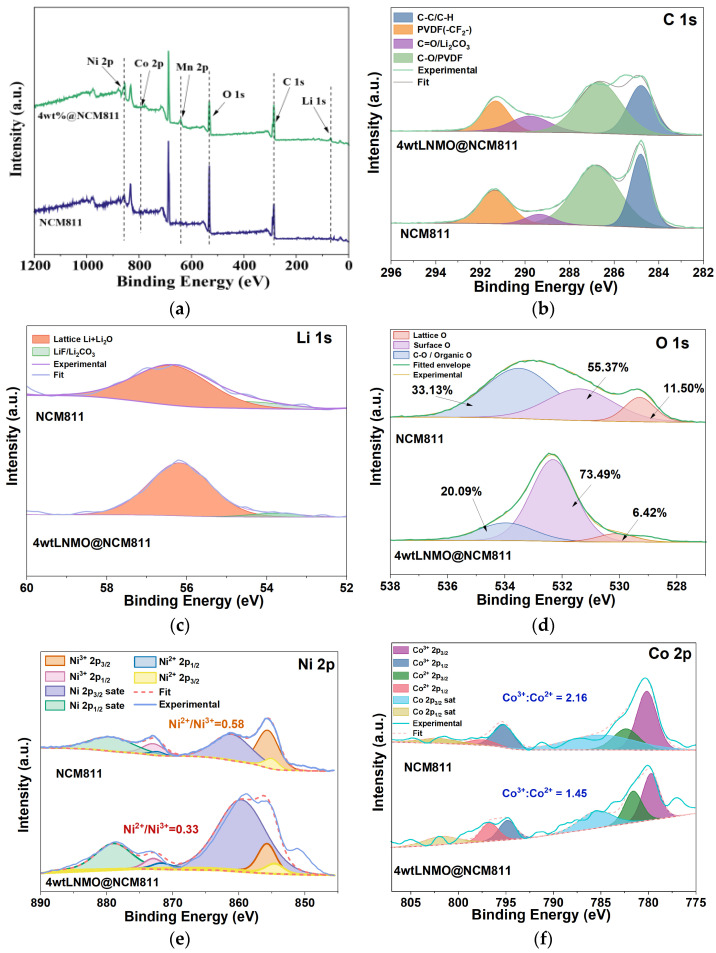
Surface chemical composition analysis via XPS. (**a**) Full survey scan spectra of pristine NCM811 and 4 wt% LNMO@NCM811; (**b**–**f**) high-resolution spectra and peak deconvolution for (**b**) C 1s, (**c**) Li 1s, (**d**) O 1s, (**e**) Ni 2p and (**f**) Co 2p.

To elucidate the surface reconstruction and interfacial chemistry induced by the LNMO coating, high-resolution XPS spectra were analyzed using a rigorous fitting procedure. All binding energies were calibrated to the C 1s peak at 284.8 eV, and peak deconvolution was performed using a Shirley background with Gaussian–Lorentzian line shapes. The survey spectra in Figure 4a confirm the presence of Ni, Co, Mn, O, C and Li signals for both electrodes, with no detectable extraneous elements, indicating that the coating procedure does not introduce observable contamination within the detection limit of XPS.

The C 1s spectra (Figure 4b) were deconvoluted into four components: C–C/C–H (284.8 eV), C–O/PVDF–CH_2_ (285.7–286.3 eV), C=O/Li_2_CO_3_ (288.5–289.0 eV), and PVDF–CF_2_ (290.8 eV) [52]. Comparing the two electrodes, the pristine NCM811 exhibits a significantly higher intensity ratio of carbonate species (C=O) associated with the decomposition of electrolyte and spontaneous surface carbonation. In contrast, the 4 wt% LNMO@NCM811 sample shows a suppressed carbonate signal, indicating a thinner and more stable CEI. This is corroborated by the Li 1s spectra (Figure 4c), where the peak associated with surface LiF/Li_2_CO_3_ (~55.4 eV) is reduced in the coated sample compared to the lattice Li peak (~54.2 eV) [53]. The O 1s spectra (Figure 4d) reveal the nature of surface oxygen species. The main peak at ~529.3 eV corresponds to lattice oxygen (M–O) in the layered structure [54]. The shoulder peaks at higher binding energies are assigned to surface-absorbed oxygen/defect species (~531.5 eV) and organic oxygen (C–O/C=O) in the CEI layer (~533.0 eV) [55]. Notably, the coated sample retains a sharper lattice oxygen feature with a reduced proportion of surface defect species (decreasing from 55.37% to 20.09%), confirming that the LNMO shell effectively protects the lattice oxygen from engaging in parasitic surface reactions. The Ni 2p spectra (Figure 4e) were fitted with two multiplets corresponding to Ni^2+^ (~854.3 eV) and Ni^3+^ (~855.8 eV), along with their characteristic satellites [56,57]. For pristine NCM811, the surface is dominated by Ni^2+^ species (Ni^2+^/Ni^3+^ ratio = 0.58), indicating severe surface reconstruction into the electrochemically inactive rock-salt phase (NiO-like). However, for the 4 wt% LNMO@NCM811, this ratio drops significantly to 0.33. This preservation of the higher valency (Ni^3+^) confirms that the spinel shell suppresses the reduction in transition metals and the formation of the rock-salt reconstruction layer. Similarly, the Co 2p spectra (Figure 4f) were deconvoluted into Co^3+^ (~780.2 eV) and Co^2+^ (~781.8 eV) components [58]. The modification in the Co^3+^/Co^2+^ ratio further reflects the alteration in the local chemical environment at the interface due to the integration of the spinel phase.

Overall, the XPS results indicate that the LNMO coating reconstructs the surface and interphase chemistry, reduces the extent of surface masking by residual species, and stabilizes the transition-metal–oxygen framework against detrimental reconstruction.

### 3.5. Electrochemical Performance

**Figure 5:**
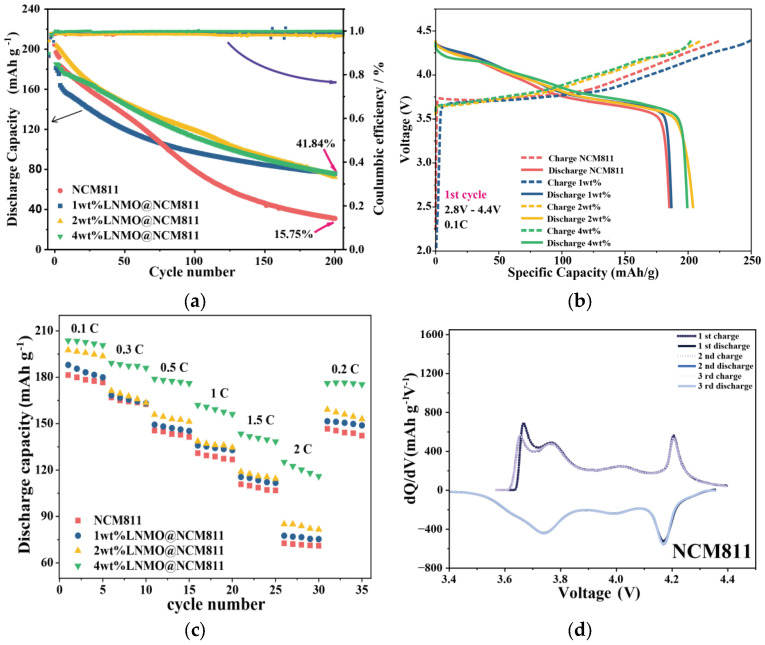

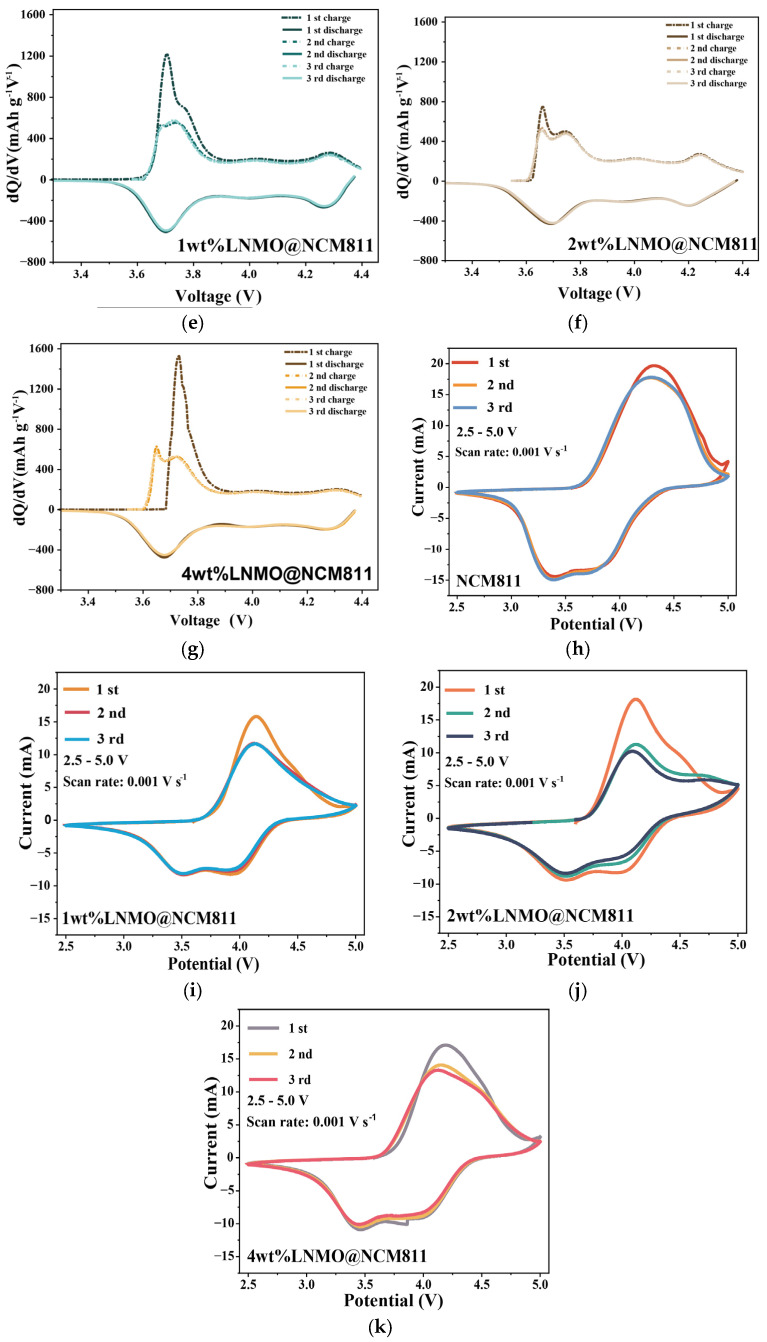
Electrochemical performance of pristine NCM811 and LNMO-coated electrodes. (**a**) Cycling performance and Coulombic efficiency at 0.5 C after formation; (**b**) first charge–discharge profiles at 0.1 C; (**c**) Rate capability from 0.1 to 2 C; (**d**–**g**) dQ/dV curves for the first three cycles of pristine NCM811, 1 wt% LNMO@NCM811, 2 wt% LNMO@NCM811 and 4 wt% LNMO@NCM811, respectively; (**h**–**k**) Cyclic voltammetry curves for the same electrodes at a scan rate of 0.001 V s^−1^.

The long-term viability of the cathodes was evaluated over a wide voltage window of 2.8–4.4 V. To ensure the reliability and reproducibility of the electrochemical results, at least five independent coin cells were assembled and tested for each sample condition. The electrochemical curves and cycling data presented in the figures correspond to the champion cell (the cell exhibiting the highest average capacity) to demonstrate the maximum potential of the material. However, performance consistency was rigorously verified across all replicates, with the standard deviation of the specific capacity calculated to be less than 3% for all tested cycles. As shown in Figure 5a, the pristine NCM811 exhibits severe capacity decay at 0.5 C, retaining only 15.75% of its initial capacity after 200 cycles. Introducing a low coating content of 1 wt% LNMO does not deliver a clear improvement in cycling stability and only marginally alters the fading trend, suggesting that a discontinuous or insufficiently protective surface layer fails to effectively mitigate degradation under high-voltage operation. Increasing the coating content to 2 wt% leads to an evident enhancement in retention, indicating that the protective effect becomes more pronounced as surface coverage improves. This trend culminates in the 4 wt% LNMO@NCM811 electrode, which achieves the highest durability and maintains 41.84% capacity retention after 200 cycles. The Coulombic efficiency remains close to unity for all samples, suggesting that the dominant capacity loss is driven by intrinsic cathode degradation and the loss of active lithium inventory, rather than by continuously intensifying parasitic reactions. The impact of the coating is further reflected in the charge–discharge profiles. In Figure 5b, the 1 wt% electrode shows larger voltage polarization in the first cycle compared with pristine NCM811, indicating increased interfacial resistance arising from an insufficient coating level. The rate capability results in Figure 5c reveal a consistent evolution with coating content. While all electrodes deliver comparable capacities at low current densities, the 1 wt% sample shows a more pronounced capacity drop as the rate increases. The 2 wt% electrode partially recovers the high-rate performance, whereas the 4 wt% LNMO@NCM811 electrode delivers the best rate capability across all tested currents. This confirms that an adequately formed shell is prerequisite to alleviating kinetic limitations and maintaining capacity at high rates. To probe the origin of these performance differences, the evolution of redox features was tracked by differential capacity analysis. The pristine NCM811 electrode shows characteristic redox peaks, but the high-voltage feature around 4.2 V displays rapid attenuation and noticeable positional shifting within the first three cycles. It is worth noting that the 1 wt% sample exhibits capacity decay rates comparable to, or even faster than, the pristine cathode. This anomaly stems from the discontinuous nature of the coating at low concentrations. Rather than forming a protective shell, the LNMO phase likely exists as isolated clusters, creating a heterogeneous surface with mismatched conductivity. This variance induces localized current hotspots and non-uniform lithium intercalation. Consequently, local regions undergo severe over-delithiation and stress accumulation at the boundaries of the spinel islands, accelerating micro-cracking and electrolyte infiltration more aggressively than the uniform degradation of the pristine surface. The severe peak distortion in the dQ/dV curves (Figure 5e) and the broadened redox separation in the CV scans (Figure 5i) for the low-loading samples confirm that surface heterogeneity may accelerate degradation. Thus, a complete and conformal shell is critical for stability. For the optimized 4 wt% LNMO@NCM811, the enhancement originates from a dual stabilization mechanism. Electrochemically, the continuous spinel shell facilitates uniform Li^+^ transport, effectively mitigating the polarization typically seen at high cutoff voltages. Mechanically, and more importantly, the robust spinel framework acts as a “clamping layer” that suppresses the abrupt anisotropic lattice contraction associated with the destructive H2→H3 phase transition near 4.2 V. This is corroborated by the dQ/dV analysis displayed in Figure 5g, where the high-voltage redox feature retains its sharpness and position over cycling, in stark contrast to the rapid decay and shifting observed in the pristine cathode. By preserving the reversibility of this critical phase transition, the cohesive coating ensures long-term structural integrity. Cyclic voltammetry provides complementary evidence for this kinetic evolution. While the pristine NCM811 in Figure 5h exhibits broad redox responses with appreciable potential separation, the 2 wt% electrode in Figure 5j begins to show improved peak shape relative to the 1 wt% sample. Finally, the 4 wt% LNMO@NCM811 electrode in Figure 5k exhibits the most distinct redox features with minimal potential separation. Crucially, aside from a slight variation in the first cycle attributed to initial activation, the subsequent scans appear nearly identical. This high degree of overlap serves as robust evidence of rapid surface stabilization and exceptional electrochemical reversibility, confirming that the continuous LNMO shell effectively mitigates the parasitic reactions that lead to voltage hysteresis. Overall, the systematic improvement from 1 wt% to 4 wt% demonstrates that sufficient coating coverage is essential to simultaneously stabilize high-voltage structural evolution and maintain favorable electrochemical kinetics. It should be noted that the performance variation across the optimized loading range is relatively subtle. However, statistical analysis based on multiple parallel cells confirms that the 4 wt% loading consistently yields the optimal electrochemical balance. Loadings below this threshold fail to provide complete surface coverage, leading to higher variance in cycle life, while loadings above 4 wt% result in a discernible increase in polarization without offering further gains in retention. Thus, the 4 wt% dosage represents the statistically meaningful “sweet spot” where the trade-off between interfacial protection and kinetic hindrance is minimized. The severe cycling deterioration of the pristine NCM811 can be directly correlated to the surface degradation features identified in the XPS analysis. Specifically, the detection of a dominant Ni^2+^ signal in the Ni 2p spectrum confirms the extensive transformation of the layered structure into a highly resistive NiO-like rock-salt phase. Concurrently, the pronounced accumulation of carbonate-rich species indicates the growth of a thick, insulating CEI layer. These degradation layers act as electronic barriers, isolating the primary particles and severing electrical contact within the secondary agglomerates. The resulting increase in inter-particle impedance and loss of active lithium inventory precipitate the rapid capacity fade and voltage polarization observed during long-term cycling.

### 3.6. Thermal Stability Analysis and Mechanism Function Fitting

**Figure 6:**
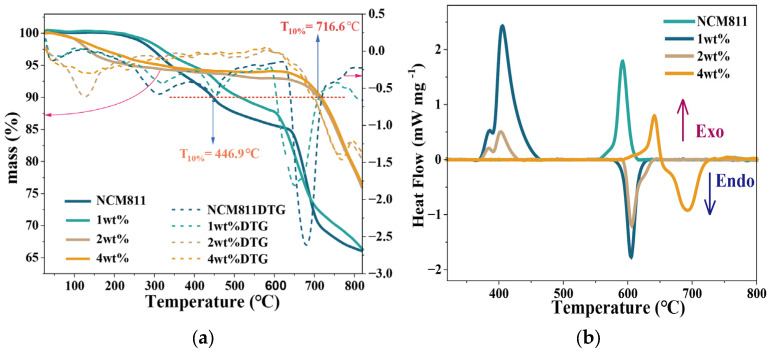

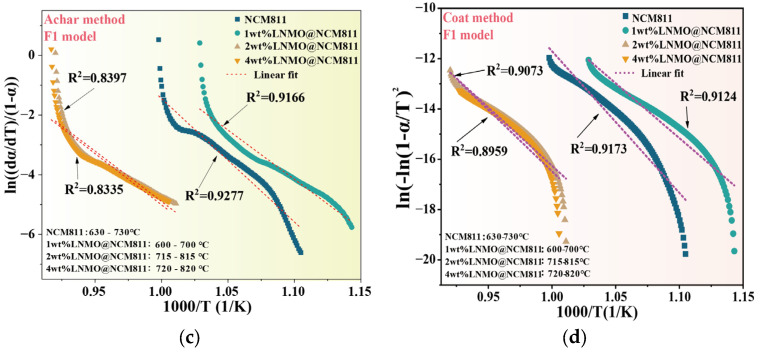
Thermal stability and kinetic analysis of the cathode materials. (**a**) TG and DTG curves; (**b**) DSC heat flow profiles; (**c**) linear fitting plots based on the Achar method; (**d**) linear fitting plots based on the Coats-Redfern method.

Thermal safety remains a critical challenge for high-energy Ni-rich cathodes [59]. To evaluate the impact of the LNMO shell, the thermal decomposition behavior of the electrodes was investigated by TG-DSC. As shown in Figure 6a, the thermogravimetric curves exhibit distinct mass-change events that are commonly associated with oxygen-related degradation and the irreversible transformation of layered oxides into rock-salt-like products at elevated temperatures. The temperature at 10% mass loss, defined as T10%, was used as a practical indicator of thermal stability. Pristine NCM811 remains stable up to 515.2 °C. Unexpectedly, the addition of a low 1 wt% LNMO loading results in a decrease of T10% to 446.9 °C. This implies a “coverage-Dependent Stability Mechanism”: an incomplete coating acts as a destabilizing factor rather than a protective barrier. The discontinuous LNMO islands create abundant semi-coherent phase boundaries with the NCM host. Under high-temperature thermal stress, these exposed interfaces likely serve as high-energy defect sites with reduced activation barriers for oxygen release. Furthermore, the mismatch in thermal expansion coefficients between the dispersed spinel islands and the layered bulk may induce localized stress concentration, effectively triggering the lattice collapse and oxygen evolution earlier than in the homogeneous pristine surface. Only when the coating coverage increases to 2 wt% and 4 wt% does the shell become sufficiently cohesive to suppress these boundary effects and impose a uniform kinetic barrier, thereby shifting the decomposition onset to significantly higher temperatures.

The heat-flow in Figure 6b provides quantitative insight into the associated heat generation. Integrating the DSC curves between 300 and 800 °C gives the exothermic and endothermic enthalpies listed in Table 3. The 1 wt% sample displays the most hazardous behavior, with a strong exothermic peak at 405.6 °C and a large exothermic enthalpy of 463.5 J g^−1^. Pristine NCM811 exhibits a smaller exotherm of 208.3 J g^−1^ with a peak at 592.2 °C, which corresponds to moderate thermal risk. In comparison, the 4 wt% LNMO@NCM811 electrode shows a much weaker exothermic signal of 81.5 J g^−1^ and a delayed peak at 641.2 °C, and a sizeable endothermic contribution of 221.7 J g^−1^ at higher temperature. The net effect is a substantial reduction in heat release and a shift in the main reaction to a higher temperature regime, which points to a safer thermal response under abuse conditions.

To clarify the kinetic origin of these differences, kinetic parameters were extracted using a combination of the differential Achar method and the integral Coats-Redfern method based on the Arrhenius equation [60]. The Achar relation can be written as

(1) $$\ln\;[\frac{d\alpha /\mathrm{dT}}{f(\alpha )}]=\ln(\frac{A}{\beta })\;-\frac{\mathrm{Ea}}{\mathrm{RT}}$$

and the Coats-Redfern equation as

(2) $$\ln\left[\frac{g(\alpha )}{T^{2}}\right]=\ln(\frac{\mathrm{AR}}{\beta \mathrm{Ea}})\;-\frac{\mathrm{Ea}}{\mathrm{RT}}$$

where *α* is the conversion degree, *β* is the heating rate, T is the absolute temperature, R is the gas constant, and f(*α*) and g(*α*) are the differential and integral forms of the reaction model [61]. Five typical mechanisms listed in Table 4 were evaluated. The linear fits in Figure 6c,d show that the first-order reaction model F1 gives the highest correlation coefficients for all samples. Although the correlation coefficients reflect the inherent complexity of the multi-step decomposition in heterogeneous cathode materials, involving simultaneous oxygen release and phase transition, the F1 model consistently provides the best fit among all tested mechanisms. The corresponding activation energies obtained from the Coats-Redfern analysis are summarized in Table 5. The 1 wt% LNMO@NCM811 electrode shows the lowest activation energy of 301.53 kJ mol^−1^, consistent with its early onset temperature and large exothermic heat. Increasing the coating level to 2 wt% and 4 wt% raises the activation energy to 334.05 and 340.12 kJ mol^−1^, respectively, suggesting a partial recovery of the kinetic barrier once a more complete coating is formed. Although pristine NCM811 exhibits the highest activation energy, it still shows a larger exothermic heat and a lower T_10%_ than the 4 wt% sample, indicating that the LNMO shell mainly improves safety by moderating the exothermic reaction pathway and delaying oxygen release rather than solely by increasing E_a_.

This study validates that a continuous LNMO spinel coating successfully reconciles the trade-off between energy density and safety in Ni-rich cathodes. Crucially, the TG-DSC and kinetic results analysis reveal that coating integrity is determinant: while a thin and discontinuous coating can aggravate the thermal instability, a sufficiently thick and uniform LNMO layer significantly delays decomposition, reduces heat release and imposes a higher kinetic barrier. This provides a wider safety margin for Ni-rich cathodes under high-temperature abuse. To underscore the efficacy of the dual-functional spinel shell, it is instructive to compare the thermal stability metrics with other surface-modified Ni-rich cathodes reported in the recent literature. Typically, inert oxide coatings such as Al_2_O_3_ and ZrO_2_ function primarily as physical barriers, raising the T_10%_ by approximately 20–40 °C relative to the pristine material (e.g., from ~220 °C to ~250 °C) [62,63]. In stark contrast, the 4 wt% LNMO@NCM811 developed in this work achieves a substantially higher T_10%_ of 716.6 °C, representing a significant enhancement. This superior stability suggests that the active spinel/layered heterostructure alters the intrinsic decomposition pathway rather than merely delaying it, offering a distinct advantage over traditional passive coating strategies.

## 4. Conclusions

This study validates that a continuous LNMO spinel coating successfully reconciles the trade-off between energy density and safety in Ni-rich cathodes. Our findings confirm that the scalable sol–gel route creates a structurally compatible heterointerface. This interface actively scavenges residual alkaline species and prevents detrimental rock-salt-like surface degradation, effectively reconditioning the surface chemistry. Mechanically, the rigid spinel shell acts as a clamp to mitigate lattice strain and suppress the abrupt H2→H3 phase transition during deep delithiation. These synergistic effects enable the optimized 4 wt% electrode to deliver superior cycling stability, retaining 41.84% of its capacity after 200 cycles compared to only 15.75% for the pristine sample. Crucially, thermal kinetic analysis further proves that the spinel layer raises the activation energy barrier to 340.1 kJ mol^−1^, shifting the critical decomposition temperatures from 515.2 °C to 716.6 °C, and significantly lowering total heat release to 81.5 J g^−1^. Beyond these technical enhancements, the use of an abundant, low-toxicity manganese-based coating—circumventing the need for additional cobalt—aligns with green manufacturing principles. This offers a sustainable, practically viable path for the gigawatt-scale deployment of safety-critical batteries in carbon-neutral applications.

## Figures and Tables

**Table 1:** Rietveld refinement parameters and micro-strain evolution of pristine and 4 wt%LNMO@ NCM811 before cycling.

Samples	*a* (Å)	*c* (Å)	*c*/*a*	*V* (Å^3^)	I(003)/I(104)	R_wp_ (%)	Micro Strain (ε × 10^−6^)
0 cycle							
NCM811	2.8719	14.1917	4.942	101.368	1.274	2.900	1161.6
4 wt%	2.8796	14.2180	4.937	102.106	1.395	2.811	1100.7

**Table 2:** Rietveld refinement parameters and micro-strain evolution of pristine and 4 wt%LNMO@ NCM811 after 200 cycles.

Samples	*a* (Å)	*c* (Å)	*c*/*a*	*V* (Å^3^)	I(003)/I(104)	R_wp_ (%)	Micro Strain (ε × 10^−6^)
200 cycles							
NCM811	2.8809	14.2178	4.935	102.195	1.12	1169.1	NCM811
4 wt%	2.8381	14.3475	5.055	100.080	1.31	1277.0	4 wt%

**Table 3:** Comparison of exothermic characteristics of pristine NCM811 and x wt% LNMO@NCM811 (x = 1, 2, 4) from DSC analysis.

Sample	Exothermic Peak (°C)	ΔH (J g^−1^)	Endothermic Peak (°C)	ΔH (J g^−1^)
NCM811	592.2 °C	208.3		
1 wt%LNMO	405.6 °C	463.5	605.4 °C	175.8
2 wt%LNMO	403.2 °C	97.0	606.2 °C	129.3
4 wt%LNMO	641.2 °C	81.5	693.5 °C	221.7

**Table 4:** Types of mechanistic model.

Number	Reaction Mechanism	F(*α*)	G(*α*)
1	First-order reaction model, n = 1.0	1 − α	−ln(1 − α)
2	Phase boundary reaction with spherical symmetry, n = 1/3	3(1 − α)^2/3^	1 − (1 − α)^1/3^
3	Jander model with two-dimensional diffusion, n = 2	(1 − α)^1/2^[1 − (1 − α)^1/2^]^−1^	[1 − (1 − α)^1/2^]^2^
4	Avrami–Erofeev model, n = 2	1/2(1 − α) [−ln(1 − α)]^−1^	[−ln(1 − α)]^2^
5	Avrami–Erofeev model, n = 3	1/3(1 − α) [−ln(1 − α)]^−2^	[−ln(1 − α)]^3^

**Table 5:** Best-fit kinetic parameters for NCM811 and x wt% LNMO@NCM811 (x = 1, 2, 4).

Sample	Method	Mechanism	E_a_ (kJ/mol)	R^2^
NCM811	Coats-Redfern	First-order reaction model, n = 1.0	395.73	0.9173
1 wt%LNMO			301.53	0.9124
2 wt%LNMO			334.05	0.9073
4 wt%LNMO			340.12	0.8959

## Data Availability

The raw data used in the analysis presented in this article will be made available by the authors upon request.

## Supplementary Materials

The following supporting information can be downloaded at: https://www.mdpi.com/article/10.3390/nano16030183/s1, Figure S1: Optical Photographs; Figure S2. SEM image and corresponding EDS elemental mapping of the pristine NCM811 sample. (a) SEM electron image of the secondary particles; (b) Carbon (C) elemental distribution map; (c) Oxygen (O) elemental distribution map; (d) Manganese (Mn) elemental distribution map; (e) Cobalt (Co) elemental distribution map; (f) Nickel (Ni) elemental distribution map; Figure S3. SEM image and corresponding EDS elemental mapping of the 4wt%LNMO@NCM811 sample. (a) SEM electron image of the secondary particles; (b) Carbon (C) elemental distribution map; (c) Oxygen (O) elemental distribution map; (d) Manganese (Mn) elemental distribution map; (e) Cobalt (Co) elemental distribution map; (f) Nickel (Ni) elemental distribution map.

## Abbreviations

The following abbreviations are used in this manuscript:

NCM811	LiNi_0.8_Co_0.1_Mn_0.1_O_2_
LNMO	LiNi_0.5_Mn_1.5_O_4_
TG-DSC	Thermogravimetric-differential scanning calorimetry
T_10%_	10% mass loss temperature
XRD	X-ray diffraction
SEM	Scanning electron microscopy
XPS	X-ray photoelectron spectroscopy
DMC	Dimethyl carbonate
PVDF	Polyvinylidene fluoride
NMP	N-methyl-2-pyrrolidone
EC	Ethylene carbonate
EMC	Ethyl methyl carbonate
