# Supplementary material for: Spinel-Encapsulated Ni-Rich Cathodes for Enhanced Thermal Safety: Unraveling the Decomposition Kinetics and Interfacial Reconstruction
Source: Nanomaterials (Basel). 2026 Jan 29;16(3):183. doi: 10.3390/nano16030183 (PMC12899607; doi:10.3390/nano16030183)
Supplement: Supplementary file 1 [file nanomaterials-16-00183-s001.zip › nanomaterials-4108911-supplementary.pdf]

Supporting Information

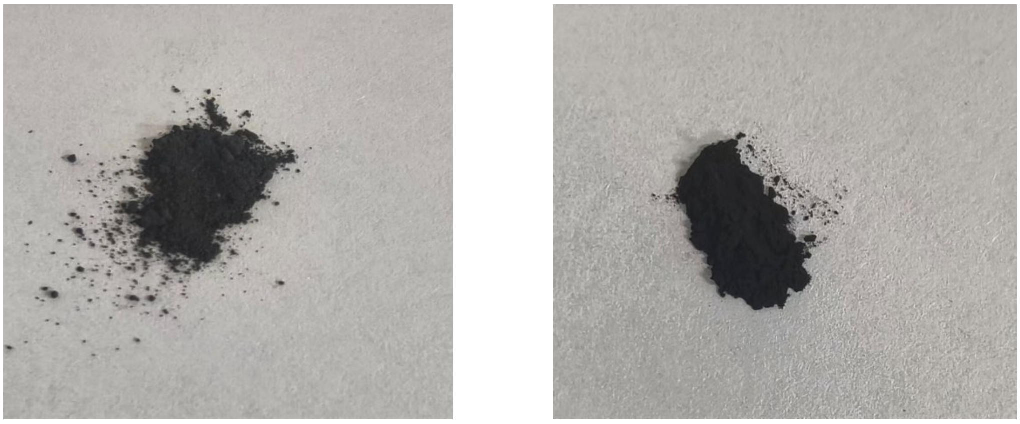

(a) LNMO@NCM811 (b) NCM811

Figure S1: Optical Photographs

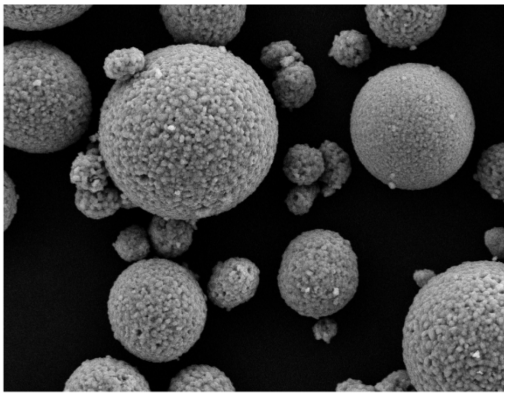

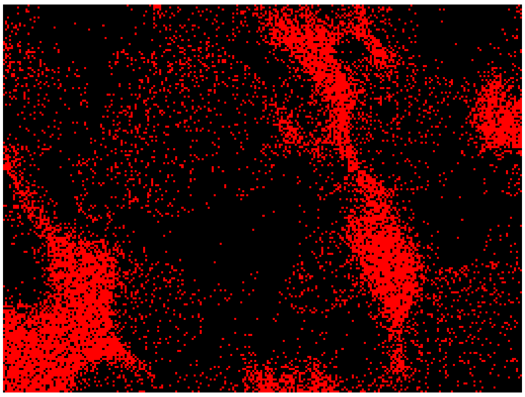

(a) (b)

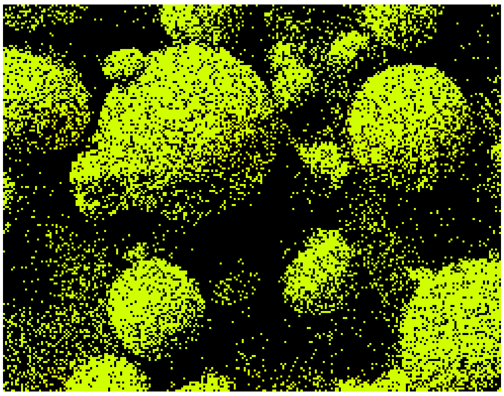

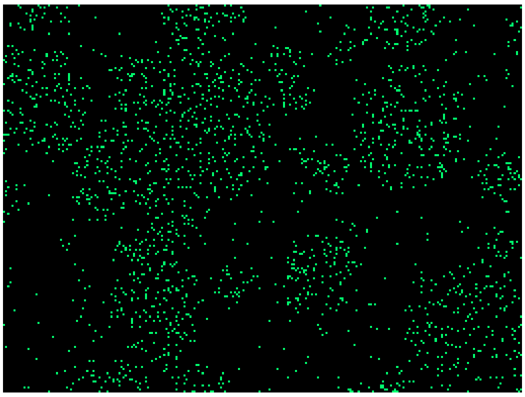

(c) (d)

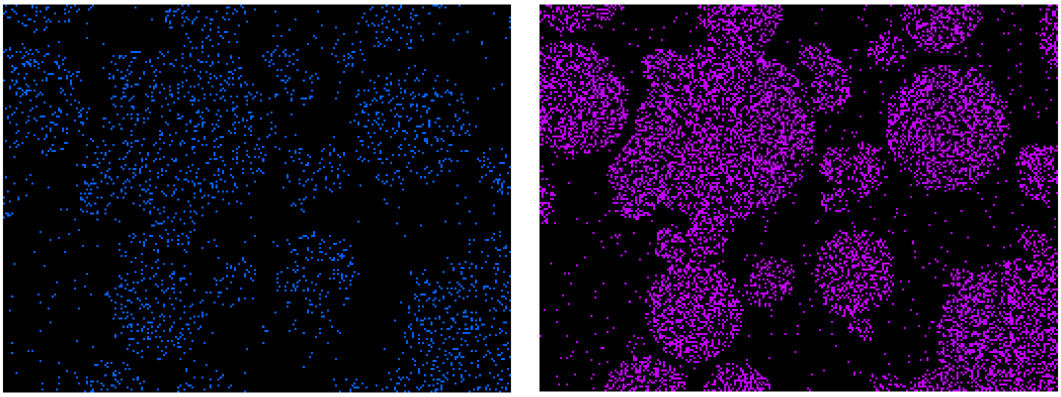

(e) (f)

Figure S2. SEM image and corresponding EDS elemental mapping of the pristine NCM811 sample. (a) SEM electron image of the secondary particles; **(b)** Carbon (C) elemental distribution map; **(c)** Oxygen (O) elemental distribution map; **(d)** Manganese (Mn) elemental distribution map; **(e)** Cobalt (Co) elemental distribution map; **(f)** Nickel (Ni) elemental distribution map.

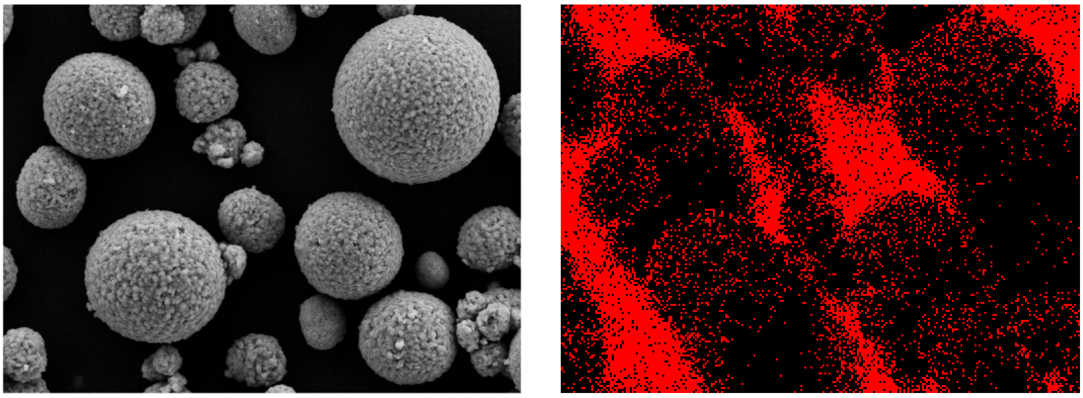

(a) (b)

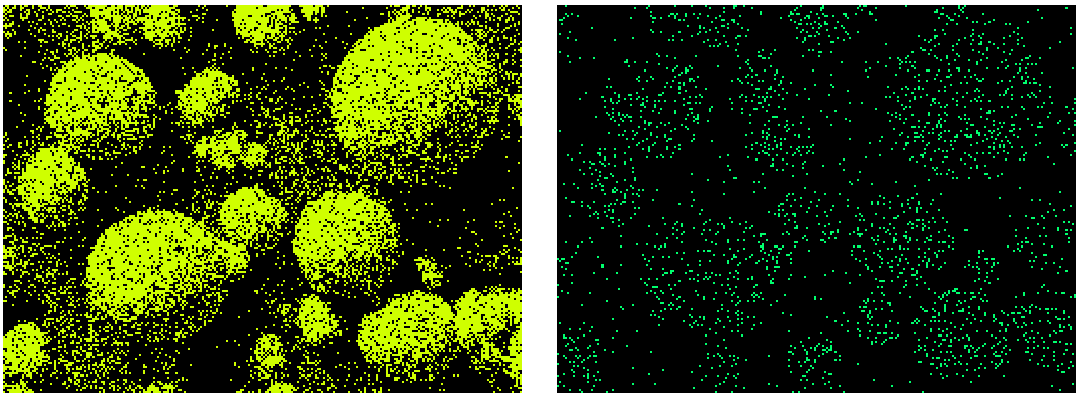

(c) (d)

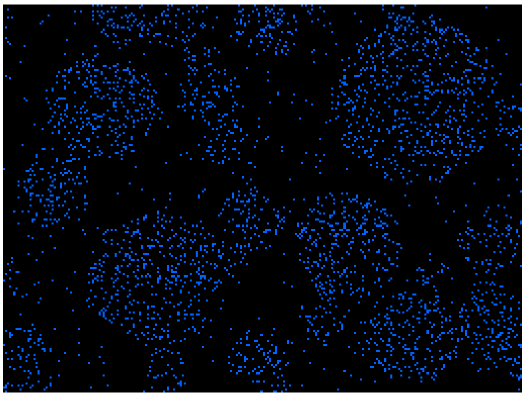

(e)

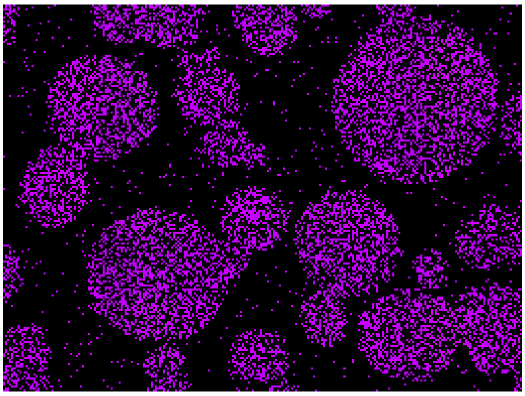

(f)

Figure S3. SEM image and corresponding EDS elemental mapping of the 4wt%LNMO@NCM811 sample. (a) SEM electron image of the secondary particles; **(b)** Carbon (C) elemental distribution map; **(c)** Oxygen (O) elemental distribution map; **(d)** Manganese (Mn) elemental distribution map; **(e)** Cobalt (Co) elemental distribution map; **(f)** Nickel (Ni) elemental distribution map.
